# Interferon gamma (IFNg) production is associated to disease parameters in TLR9-induced secondary hemophagocytic lymphohistiocytosis (sHLH) in mice

**DOI:** 10.1186/1546-0096-12-S1-O2

**Published:** 2014-09-17

**Authors:** Cristina De Min, Vanessa Buatois, Laurence Chatel, Laura Cons, Marie Kosco-Vilbois, Walter Ferlin

**Affiliations:** 1 Clinical Development, NovImmune SA, Plan-Les-Ouates Geneve, Switzerland; 2 Pre-clinical Development, NovImmune SA, Plan-Les-Ouates Geneve, Switzerland

## Introduction

IFNg is the pivotal mediator in the murine model of primary HLH. Mice given repeated injection with the TLR9 agonist, CpG-containing oligodeoxynucleotides, develop pathology resembling the human disease, sHLH. Coadministration of an anti-IL-10 Receptor (R) monoclonal antibody (mAb) with CpG induces more severe disease characterized also by hemophagocytosis (fulminant sHLH).

## Objectives

We evaluated whether the neutralization of IFNg in murin sHLH and fulminant sHLH affected the disease features and we investigated whether treatment with an anti-IFNg mAb affected the clinical and laboratory features in murine models of sHLH and fulminant sHLH; we also exploited the *in vivo* principle that, in the presence of an anti-IFNg antibody, circulating IFNg bound to the antibody is incorporated in a complex leading to accumulation of the cytokine in serum, therefore allowing the quantification of IFNg production.

## Methods

C57BL/6 mice received i.p. injections of CpG on days 0, 2, 4, 7 & 9. Neutralizing IL-10R, mAb 1B1.3A at 200 µg/mouse (days 0,2,4,6), and anti-mouse IFNg, mAb XMG1.2 at 100 mg/kg (days 1, 3, 6) were administered *i.v*..

## Results

In murine sHLH, the neutralization of IFNg caused a reduction in body weight loss and splenomegaly, normalized white blood cell counts and hyperferritinemia, and corrected anemia. Blockade of IFNg in mice with fulminant sHLH improved key disease features by decreasing the body weight loss by 20%, reduced splenomegaly by 23%, improved anemic parameters by 13%, reversed cytopenia by 30% and normalized sHLH-associated cytokine storm as evidenced by a 60% decrease in circulating levels of TNFα. Circulating levels of IFNg reached steady state at 250 ng/ml in both models (sHLH and fulminant sHLH). Expression of IFNg-induced inflammatory genes demonstrated that spleen and liver are major sites of IFNg production.

## Conclusion

Neutralization of IFNg appears to effectively improve the clinical and laboratory features in the CpG-induced models of sHLH, including fulminant sHLH. These data offer a rationale for the neutralization of IFNg as a potential targeted therapeutic approach in patients with severe form of sHLH.

## Disclosure of interest

None declared.

